# A Genome-Wide Association Study Identifies Five Loci Influencing Facial Morphology in Europeans

**DOI:** 10.1371/journal.pgen.1002932

**Published:** 2012-09-13

**Authors:** Fan Liu, Fedde van der Lijn, Claudia Schurmann, Gu Zhu, M. Mallar Chakravarty, Pirro G. Hysi, Andreas Wollstein, Oscar Lao, Marleen de Bruijne, M. Arfan Ikram, Aad van der Lugt, Fernando Rivadeneira, André G. Uitterlinden, Albert Hofman, Wiro J. Niessen, Georg Homuth, Greig de Zubicaray, Katie L. McMahon, Paul M. Thompson, Amro Daboul, Ralf Puls, Katrin Hegenscheid, Liisa Bevan, Zdenka Pausova, Sarah E. Medland, Grant W. Montgomery, Margaret J. Wright, Carol Wicking, Stefan Boehringer, Timothy D. Spector, Tomáš Paus, Nicholas G. Martin, Reiner Biffar, Manfred Kayser

**Affiliations:** 1 Department of Forensic Molecular Biology, Erasmus MC University Medical Center Rotterdam, Rotterdam, The Netherlands; 2 Department of Medical Informatics, Erasmus MC University Medical Center Rotterdam, Rotterdam, The Netherlands; 3 Department of Radiology, Erasmus MC University Medical Center Rotterdam, Rotterdam, The Netherlands; 4 Interfaculty Institute for Genetics and Functional Genomics, Ernst-Moritz-Arndt University Greifswald, Greifswald, Germany; 5 Queensland Institute of Medical Research, Brisbane, Australia; 6 Rotman Research Institute, University of Toronto, Toronto, Canada; 7 Centre for Addiction and Mental Health, University of Toronto, Toronto, Canada; 8 Department of Twin Research and Genetic Epidemiology, King's College London, London, United Kingdom; 9 Department of Epidemiology, Erasmus MC University Medical Center Rotterdam, Rotterdam, The Netherlands; 10 Department of Internal Medicine, Erasmus MC University Medical Center Rotterdam, Rotterdam, The Netherlands; 11 Department of Imaging Science and Technology, Faculty of Applied Sciences, Delft University of Technology, Delft, The Netherlands; 12 Centre for Advanced Imaging, University of Queensland, Brisbane, Australia; 13 Laboratory of Neuroimaging, School of Medicine, University of California Los Angeles, Los Angeles, California, United States of America; 14 Center of Oral Health, Department of Prosthodontics, Gerostomatology, and Dental Materials, University Medicine Greifswald, Greifswald, Germany; 15 Department of Radiology and Neuroradiology, University Medicine Greifswald, Greifswald, Germany; 16 The Hospital of Sick Children, University of Toronto, Toronto, Canada; 17 Institute for Molecular Bioscience, University of Queensland, Brisbane, Australia; 18 Department of Medical Statistics and Bioinformatics, Leiden University Medical Center, Leiden, The Netherlands; 19 Montréal Neurological Institute, McGill University, Montréal, Canada; Georgia Institute of Technology, United States of America

## Abstract

Inter-individual variation in facial shape is one of the most noticeable phenotypes in humans, and it is clearly under genetic regulation; however, almost nothing is known about the genetic basis of normal human facial morphology. We therefore conducted a genome-wide association study for facial shape phenotypes in multiple discovery and replication cohorts, considering almost ten thousand individuals of European descent from several countries. Phenotyping of facial shape features was based on landmark data obtained from three-dimensional head magnetic resonance images (MRIs) and two-dimensional portrait images. We identified five independent genetic loci associated with different facial phenotypes, suggesting the involvement of five candidate genes—*PRDM16*, *PAX3*, *TP63*, *C5orf50*, and *COL17A1*—in the determination of the human face. Three of them have been implicated previously in vertebrate craniofacial development and disease, and the remaining two genes potentially represent novel players in the molecular networks governing facial development. Our finding at *PAX3* influencing the position of the nasion replicates a recent GWAS of facial features. In addition to the reported GWA findings, we established links between common DNA variants previously associated with NSCL/P at 2p21, 8q24, 13q31, and 17q22 and normal facial-shape variations based on a candidate gene approach. Overall our study implies that DNA variants in genes essential for craniofacial development contribute with relatively small effect size to the spectrum of normal variation in human facial morphology. This observation has important consequences for future studies aiming to identify more genes involved in the human facial morphology, as well as for potential applications of DNA prediction of facial shape such as in future forensic applications.

## Author Summary

Monozygotic twins look more alike than dizygotic twins or other siblings, and siblings in turn look more alike than unrelated individuals, indicating that human facial morphology has a strong genetic component. We quantitatively assessed human facial shape phenotypes based on statistical shape analyses of facial landmarks obtained from three-dimensional magnetic resonance images of the head. These phenotypes turned out to be highly promising for studying the genetic basis of human facial variation in that they showed high heritability in our twin data. A subsequent genome-wide association study (GWAS) identified five candidate genes affecting facial shape in Europeans: *PRDM16*, *PAX3*, *TP63*, *C5orf50*, and *COL17A1*. In addition, our data suggest that genetic variants associated with NSCL/P also influence normal facial shape variation. Overall, this study provides novel and confirmatory links between common DNA variants and normal variation in human facial morphology. Our results also suggest that the high heritability of facial phenotypes seems to be explained by a large number of DNA variants with relatively small individual effect size, a phenomenon well known for other complex human traits, such as adult body height.

## Introduction

The morphogenesis and patterning of the face is one of the most complex events in mammalian embryogenesis. Signaling cascades initiated from both facial and neighboring tissues mediate transcriptional networks that act to direct fundamental cellular processes such as migration, proliferation, differentiation and controlled cell death. The complexity of human facial development is reflected in the high incidence of congenital craniofacial anomalies, and almost certainly underlies the vast spectrum of subtle variation that characterizes facial appearance in the human population.

Facial appearance has a strong genetic component; monozygotic (MZ) twins look more similar than dizygotic (DZ) twins or unrelated individuals. The heritability of craniofacial morphology is as high as 0.8 in twins and families [1], [2], [3]. Some craniofacial traits, such as facial height and position of the lower jaw, appear to be more heritable than others [1], [2], [3]. The general morphology of craniofacial bones is largely genetically determined and partly attributable to environmental factors [4]–[11]. Although genes have been mapped for various rare craniofacial syndromes largely inherited in Mendelian form [12], the genetic basis of normal variation in human facial shape is still poorly understood. An appreciation of the genetic basis of facial shape variation has far reaching implications for understanding the etiology of facial pathologies, the origin of major sensory organ systems, and even the evolution of vertebrates [13], [14]. In addition, it is feasible to speculate that once the majority of genetic determinants of facial morphology are understood, predicting facial appearance from DNA found at a crime scene will become useful as investigative tool in forensic case work [15]. Some externally visible human characteristics, such as eye color [16]–[18] and hair color [19], can already be inferred from a DNA sample with practically useful accuracies.

In a recent candidate gene study carried out in two independent European population samples, we investigated a potential association between risk alleles for non-syndromic cleft lip with or without cleft palate (NSCL/P) and nose width and facial width in the normal population [20]. Two NSCL/P associated single nucleotide polymorphisms (SNPs) showed association with different facial phenotypes in different populations. However, facial landmarks derived from 3-Dimensional (3D) magnetic resonance images (MRI) in one population and 2-Dimensional (2D) portrait images in the other population were not completely comparable, posing a challenge for combining phenotype data. In the present study, we focus on the MRI-based approach for capturing facial morphology since previous facial imaging studies by some of us have demonstrated that MRI-derived soft tissue landmarks represent a reliable data source [21], [22].

In geometric morphometrics, there are different ways to deal with the confounders of position and orientation of the landmark configurations, such as (1) superimposition [23], [24] that places the landmarks into a consensus reference frame; (2) deformation [25]–[27], where shape differences are described in terms of deformation fields of one object onto another; and (3) linear distances [28], [29], where Euclidean distances between landmarks instead of their coordinates are measured. Rationality and efficacy of these approaches have been reviewed and compared elsewhere [30]–[32]. We briefly compared these methods in the context of our genome-wide association study (GWAS) (see Methods section) and applied them when appropriate.

We extracted facial landmarks from 3D head MRI in 5,388 individuals of European origin from Netherlands, Australia, and Germany, and used partial Procrustes superimposition (PS) [24], [30], [33] to superimpose different sets of facial landmarks onto a consensus 3D Euclidean space. We derived 48 facial shape features from the superimposed landmarks and estimated their heritability in 79 MZ and 90 DZ Australian twin pairs. Subsequently, we conducted a series of GWAS separately for these facial shape dimensions, and attempted to replicate the identified associations in 568 Canadians of European (French) ancestry with similar 3D head MRI phenotypes and additionally sought supporting evidence in further 1,530 individuals from the UK and 2,337 from Australia for whom facial phenotypes were derived from 2D portrait images.

## Results

**Figure 1:**
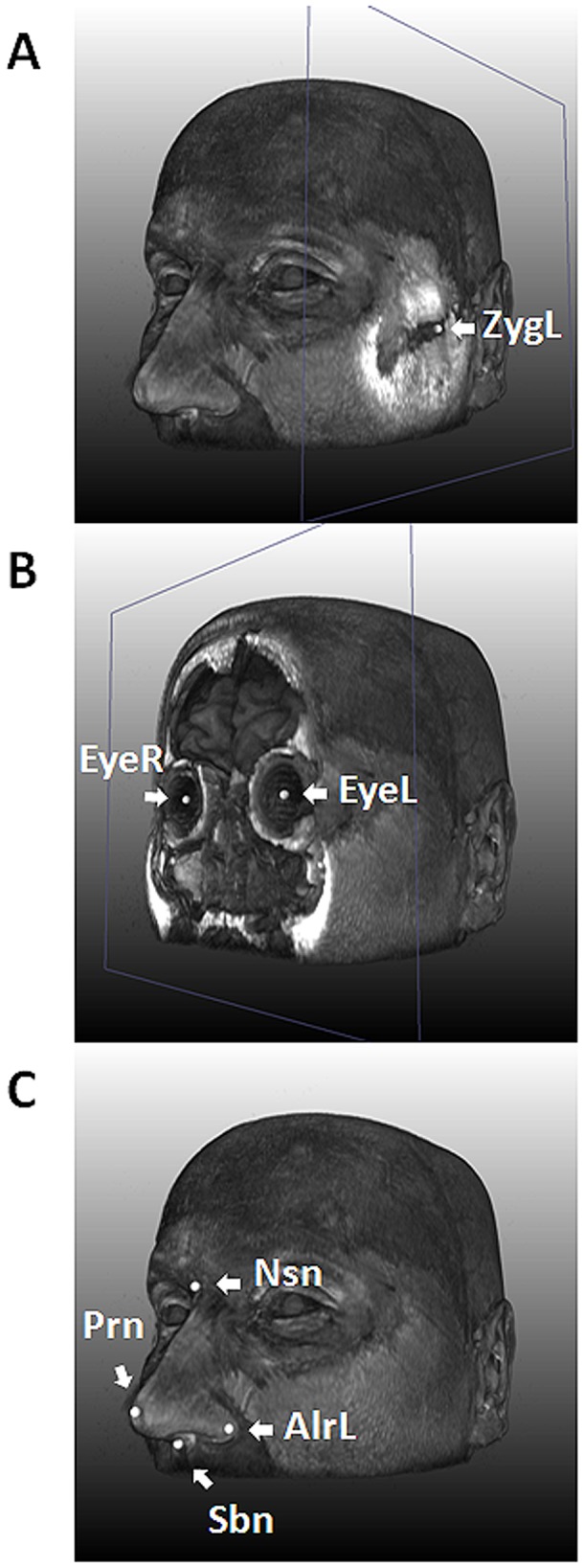
Nine facial landmarks extracted via image registration tools from 3D MRIs. An MRI of one of the authors (MK) is used for illustration. A, with the landmark for left zygion (ZygL) highlighted, where a clipping plane was used to uncover the bone; B, with the landmarks for left (EyeL) and right pupils (EyeR) highlighted, where a clipping plane was used to uncover the vitreous humor; C, with the four nasal landmarks highlighted, including the left alare, nasion (Nsn), pronasale (Prn), and subnasale (Sbn).

Characteristics of the study cohorts from The Netherlands (RS1, RS2), Australia (QTIMS, BLTS), Germany (SHIP, SHIP-TREND), Canada (SYS) and the United Kingdom (TwinsUK) are provided in Table 1. All participants included in this study were of European ancestry. Facial landmarks in the discovery cohorts RS1, RS2, QTIMS, SHIP, and SHIP-TREND were derived from directly comparable 3D head MRIs analyzed using the very same method (Figure 1). Similar 3D MRIs were available in SYS but the phenotyping method used here was slightly different (see Method). For the BLTS and TwinsUK cohorts, facial landmarks were derived from 2D portrait photos. The SYS (mean age 15 years), QTIMS (mean age 23 years), and BLTS (mean age 23 years) cohorts were on average much younger than other cohorts considered (mean age over 50 years, Table 1). The majority of the TwinsUK cohort was female (95.5%).

**Table 1:** Characteristics of the study subjects (N = 9,823).

Cohort	Country	Individual	For	Image	N	Male%	Age	±	sd
RS1	Netherlands	Unrelated	discovery	3D head MRI	2,470	46.4	59.7	±	8.0
RS2	Netherlands	Unrelated	discovery	3D head MRI	745	43.1	59.0	±	7.9
QTIMS	Australia	Twins	discovery	3D head MRI	545	39.6	23.7	±	2.3
SHIP	Germany	Unrelated	discovery	3D head MRI	797	47.3	46.0	±	12.8
SHIP-TREND	Germany	Unrelated	discovery	3D head MRI	831	44.8	50.4	±	13.6
SYS	Canada	Siblings	replication	3D head MRI	568	48.1	15.1	±	1.9
TwinsUK	UK	Twins	replication	2D portrait photo	1,530	9.5	58.4	±	12.9
BLTS	Australia	Twins	replication	2D portrait photo	2,337	47.8	23.6	±	4.6

**Figure 2:**
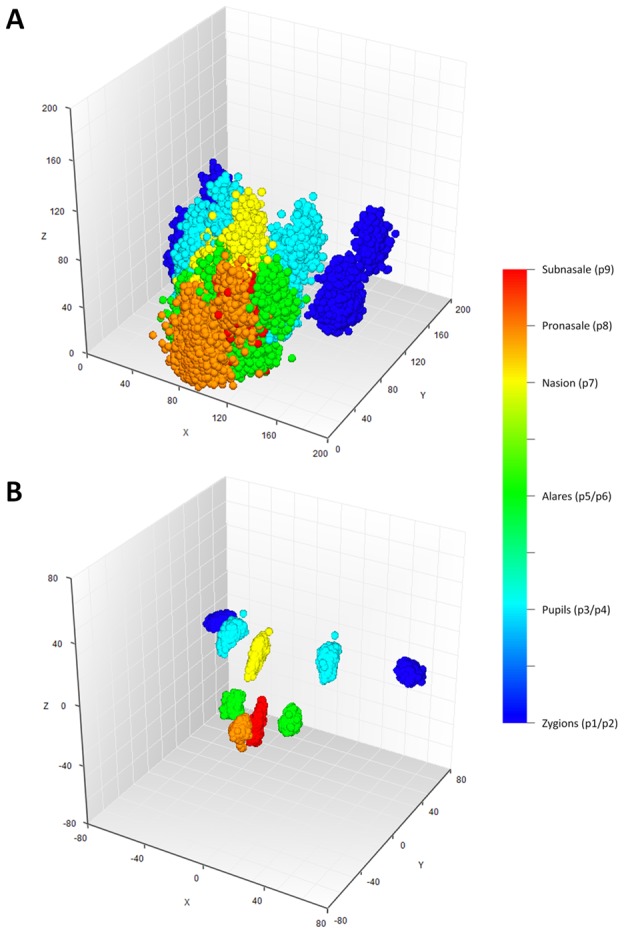
Facial landmarks from 3D MRI in all 5,388 individuals from the discovery cohorts RS1, RS2, QTIMS, SHIP, and SHIP-TREND. A, all raw landmarks before un-scaled PS; and B, after un-scaled PS.

For 3D MRI based phenotyping, we focused on the nine most well-defined landmarks from the upper part of the face, including Zygion Right (ZygR), Zygion Left (ZygL), Eyeball Right (EyeR), Eyeball Left (EyeL), Alare Right (AlrR), Alare Left (AlrL), Nasion (Nsn), Pronasale (Prn), and Subnasale (Sbn) (Figure 1). The lower part of the face i.e., from underneath the nose further down was not available due to brain-focussed MRI scanning. Raw landmark coordinates from 5,388 individuals in the five discovery cohorts (RS1, RS2, QTIMS, SHIP, and SHIP-TREND) showed systematic differences in position and orientation (Figure 2A). They were superimposed onto a consensus 3D Euclidean space based on partial PS (Figure 2B). A total of 27 principal components (PCs), and the centroid size parameter, were derived from the superimposed landmarks. Eleven PCs, each explaining >1% and all together explaining 96.0% of the total shape variance, were selected for further genetic association analysis (Table S1). Furthermore, we derived 36 Euclidean distances between each pair of landmarks. The partial PS had no effect on inter-landmark distances i.e., the distances remain the same after the superimposition. We derived the phenotypic correlations in discovery cohorts containing only adults or young adults. The SYS cohort was excluded from this correlation analysis because the changes through adolescence may confound the effect of age. Centroid size was highly correlated with the first PC (*r* = 0.96, Table S1) as well as with all 36 inter-landmark distances (mean *r* = 0.76; minimal *r* = 0.56 for Prn-Sbn; maximal *r* = 0.94 for ZygR-AlrL; Table S2). Inter-landmark distances were also correlated with each other (mean *r* = 0.56; minimal *r* = 0.10 between EyeL-AlrL and ZygL-EyeL; maximal *r* = 0.96 between AlrR-Nsn and AlrL-Nsn; Table S2). The distances between symmetric landmarks all showed the highest correlations (Table S2), consistent with general knowledge about facial symmetry. Compared with females, males on average had greater centroid size (P<1.0×10^−300^) and on average 5 mm larger inter-landmark distances (Table S3). These values are similar to the sex-specific ranges previously reported from cranial data [4]. After adjusting for the effect of centroid size, the most characteristic sex effect was that males on average had larger noses than females (AlrL-Prn and AlrR-Prn; 4 mm difference; P<1.0×10^−141^; Table S3). This sex difference is also illustrated using a thin plate splines deformation (Figure S1), showing a larger nose size in males (Figure S1C). Increased age was most significantly associated with increased bizygomatic distance (ZygR-ZygL, P = 1.9×10^−111^, Table S3). This is unlikely to be explained by the amount of subcutaneous fat in elderly people since the zygion landmarks were placed on the cortex of the bone. Heritability of 36 inter-landmark distances was estimated in 79 MZ and 90 DZ Australian twins (range 0.46–0.79, mean 0.67; Table S4). The phenotypic correlations in MZ pairs (*r* = 0.71) were on average much higher than those in DZ pairs (*r* = 0.28; Table S4). These estimates are consistent with previous facial morphology studies [1], [2] and suggest reasonably high reliability of the derived phenotypes.

**Figure 3:**
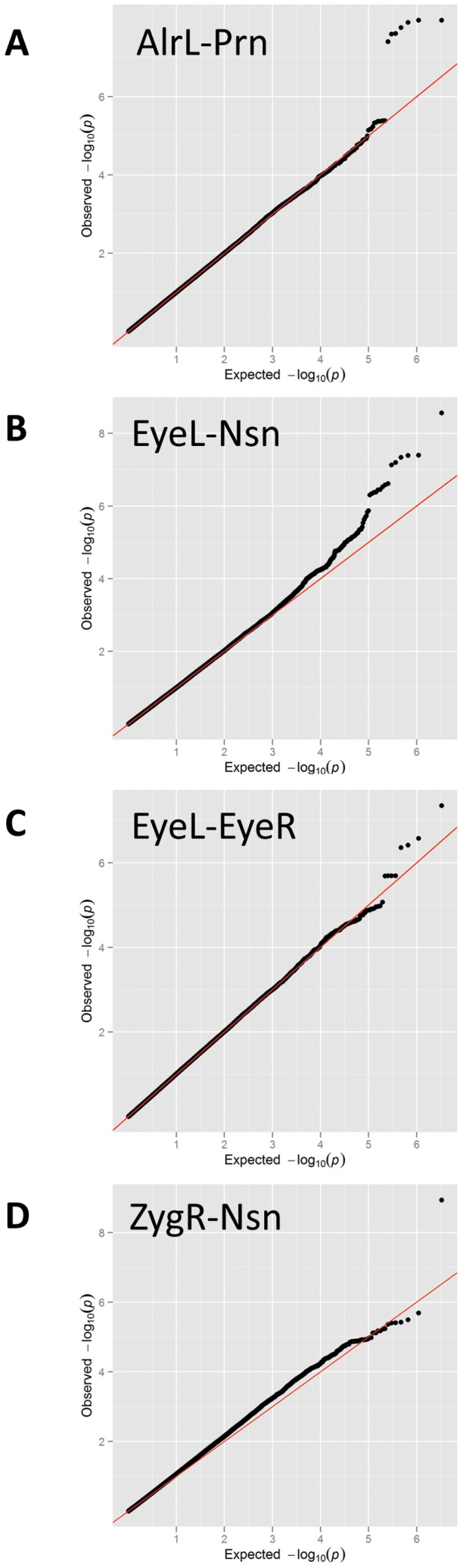
Quantile-Quantile (Q-Q) plots for the GWAS. Quantile-Quantile plots for the GWAS of (A) AlrL-Prn, (B) EyeL-Nsn, (C) EyeL-EyeR, and (D) ZygR-Nsn.

**Figure 4:**
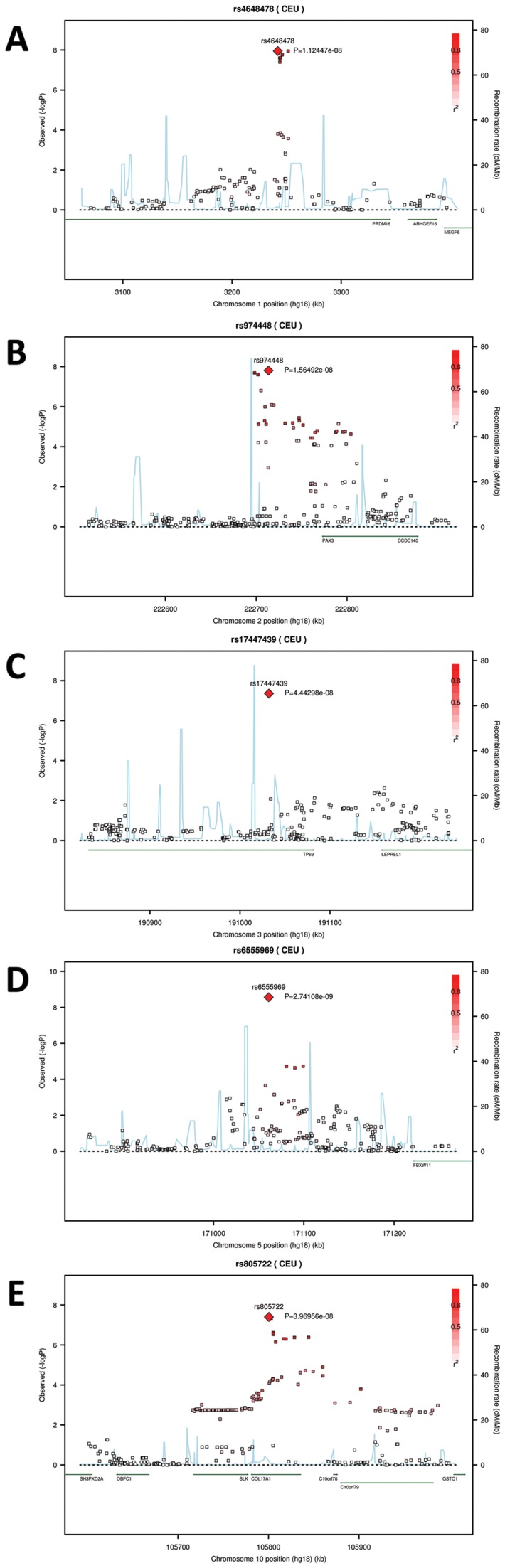
Five genomic regions harboring SNPs reaching genome-wide significant associations with facial shape phenotypes in a meta-analysis of five GWAS in discovery cohorts. The association signals (the −log10 P-values) are plotted against physical positions of each SNP in a 400 kb region centered by the most significantly associated SNP (NCBI build 36.3). Known genes in the region are aligned at the bottom. A. 1p36.23-p33 associated with AlrL-Prn, candidate gene *PRDM16*; B. 2q35 associated with EyeR-Nsn, candidate gene *PAX3*; C. 3q28 associated with EyeR-EyeL, candidate gene *TP63*. D. 5q35.1 associated with EyeL-Nsn, candidate gene *C5orf50*; E. 10q24.associated with EyeL-Nsn, candidate gene *COL17A1*.

We conducted a discovery phase GWAS in the combined sample (N = 5,388) from RS1, RS2, QTIMS, SHIP, and SHIP-TREND where facial shape phenotypes were all derived from comparable 3D head MRIs and using the same approach. We tested 2,558,979 autosomal SNPs for association with 48 facial phenotypes, including the centroid size, 36 inter-landmark Euclidean distances, and 11 shape PCs. Q-Q plots (Figure 3) and genomic inflation factors (λ<1.03) did not show any sign of inflation of the test statistics. Since many phenotypes tested were highly correlated (Table S1, Table S2), and Bonferroni correction of 48 traits would obviously be too stringent, we considered the traditional threshold P<5×10^−8^ as the significance threshold in the discovery phase. The GWAS revealed five independent loci at P<5×10^−8^ (Table 2). All these signals were observed for inter-landmark distances and most involved the nasion landmark. No genome-wide significant associations were found for individual PCs or for the centroid size. The genome-wide significantly associated SNPs were located either within (missense or intronic) or very close to (<10 kb) the following five genes: *PRDM16*, *PAX3*, *TP63*, *C5orf50*, and *COL17A1*. Notably, our finding at *PAX3* reflects an independent discovery from a parallel GWAS of facial features recently reported by Paternoster et al. [34], which identified an intronic SNP of *PAX3*, rs7559271, in association with the nasion position. In our study, three different SNPs, rs16863422, rs12694574, and rs974448 at *PAX3* on chromosome 2q35, in the same linkage-disequilibrium (LD) block containing rs7559271, were associated with the distance between the eyeballs and nasion (7.1×10^−7^<P<1.6×10^−8^ for EyeR-Nsn and EyeL-Nsn; Table 2, Figure 4B). The SNP rs7559271 from Paternoster et al. was nominally significantly associated with EyeR-Nsn (P = 0.008) and EyeL-Nsn (P = 0.004) in our data. We therefore independently confirm a role for *PAX3* in contributing to facial shape variation at the genome-wide scale, which provides confidence in the remainder of our GWAS findings. Multiple intronic SNPs of *PRDM16* on chromosome 1p36.23-p33 were associated with nose width and nose height (such as rs4648379, 2.5×10^−7^<P<1.1×10^−8^ for AlrL-Prn and AlrR-Prn; Table 2, Figure 4A). An intronic SNP of *TP63* on chromosome 3q28, rs17447439, showed association with the distance between eyeballs (P = 4.4×10^−8^ for EyeR-EyeL, Table 2, Figure 4C). A SNP rs6555969 very close to *C5orf50* on chromosome 5q35.1 was associated with nasion position (5.8×10^−7^<P<1.2×10^−9^ for ZygR-Nsn, ZygL-Nsn, EyeR-Nsn, and EyeL-Nsn; Table 2, Figure 4D). A missense SNP rs805722 in *COL17A1* on chromosome 10q24.3 was also associated with the distance between eyeballs and nasion (6.5×10^−7^<P<4.0×10^−8^ for EyeR-Nsn and EyeL-Nsn; Table 2, Figure 4E).

**Table 2:** SNPs associated with facial shape features from discovery GWAS and their replications.

								Discovery (N = 5,388)				SYS (N = 568)					BLTS+TwinSUK (N = 3,867)			
Gene	SNP	Chr	BP	Eff	Alt	FreqEff	Trait*	Beta	Se	P		Beta	se	P	%		Beta	se	P	%
*PRDM16*	rs4648379	1p36.23-p33	3251376	T	C	0.28	AlrL-Prn	−0.26	0.05	1.13E-08		0.02	0.21	0.930	1.1		0.13	0.09	0.152	0.0
							AlrR-Prn	−0.24	0.05	2.50E-07		−0.04	0.22	0.841			0.15	0.09	0.096	
*PAX3*	rs974448	2q35	222713558	G	A	0.17	EyeR-Nsn	0.29	0.05	1.56E-08		−0.19	0.20	3.6E-01	1.0		0.10	0.13	0.438	3.6
							EyeL-Nsn	0.29	0.05	7.06E-08		0.06	0.14	6.6E-01			0.21	0.12	0.076	
*TP63*	rs17447439	3q28	191032117	G	A	0.04	EyeR-EyeL	-0.91	0.15	4.44E-08		−0.42	0.68	5.4E-01	6.4		−0.56	0.27	**0.043**	21.4
*C5orf50*	rs6555969	5q35.1	171061069	T	C	0.33	ZygR-Nsn	0.41	0.07	1.17E-09		0.31	0.14	**3.2E-02**	16.0		—	—	—	17.9
							ZygL-Nsn	0.35	0.07	5.80E-07		0.39	0.14	**5.6E-03**			—	—	—	
							EyeR-Nsn	0.24	0.04	2.05E-08		0.42	0.12	**3.7E-04**			0.06	0.10	0.590	
							EyeL-Nsn	0.26	0.04	2.28E-09		0.47	0.12	**7.5E-05**			0.21	0.10	**0.031**	
*COL17A1*	rs805722	10q24.3	105800390	T	C	0.19	EyeL-Nsn	0.29	0.05	3.97E-08		0.54	0.16	**5.9E-04**	18.1		0.08	0.10	0.510	0.0
							EyeR-Nsn	0.26	0.05	6.47E-07		0.51	0.15	**9.7E-04**			−0.23	0.13	0.074	

SNPs with P<5e-8 in discovery phase are shown, one SNP per loci; symmetric facial features are shown for both when one is involved.

FreqEff, the overall frequency of the effect allele in all cohorts.

Units in the discovery and SYS cohorts are in millimeters.

% in SYS, the percentage of P values<0.05 for testing 1,540 pair-wise distances between 56 landmarks.

% in BLTS+TwinsUK, the percentage of P values<0.05 for testing 28 pair-wise distances between 8 landmarks.

* Zygions could not be reliably derived from BLTS and TwinsUK 2D portrait photos.

We attempted to replicate our GWAS findings in the SYS cohort (N = 568). Unlike the other (adult) cohorts, the SYS cohort is an adolescent one, with a mean age of 15 and a minimum age of 12 years. This may potentially lead to false negative replications since the face continues to develop and ossify throughout adolescence in a different manner than in the adult, especially in male adolescents [22]. On the other hand, the recent identification of *PAX3* [34] was based on an adolescent cohort. Our independent identification of *PAX3* in adults here demonstrates that at least some genetic effects on facial features that manifest in adolescence remain detectable in adulthood. The association signal at *PAX3*, however, did not replicate in SYS, possibly due to the small sample size available in this replication cohort. Likewise, the signals at *PRDM16* and *TP63* were not replicated in SYS. Two other loci, *C5orf50* and *COL17A1*, were successfully replicated for the same phenotypes (0.032<P<7.5×10^−5^ for *C5orf50* and 9.7×10^−4^<P<5.9×10^−4^ for *COL17A1*; Table 2). Besides the exact replication, association signals at *C5orf50* and *COL17A1* were observed for multiple phenotypes, i.e. 16.0% and 18.1% of the 1,540 pair-wise distances between all 56 landmarks available in SYS [22] (Table 2).

**Figure 5:**
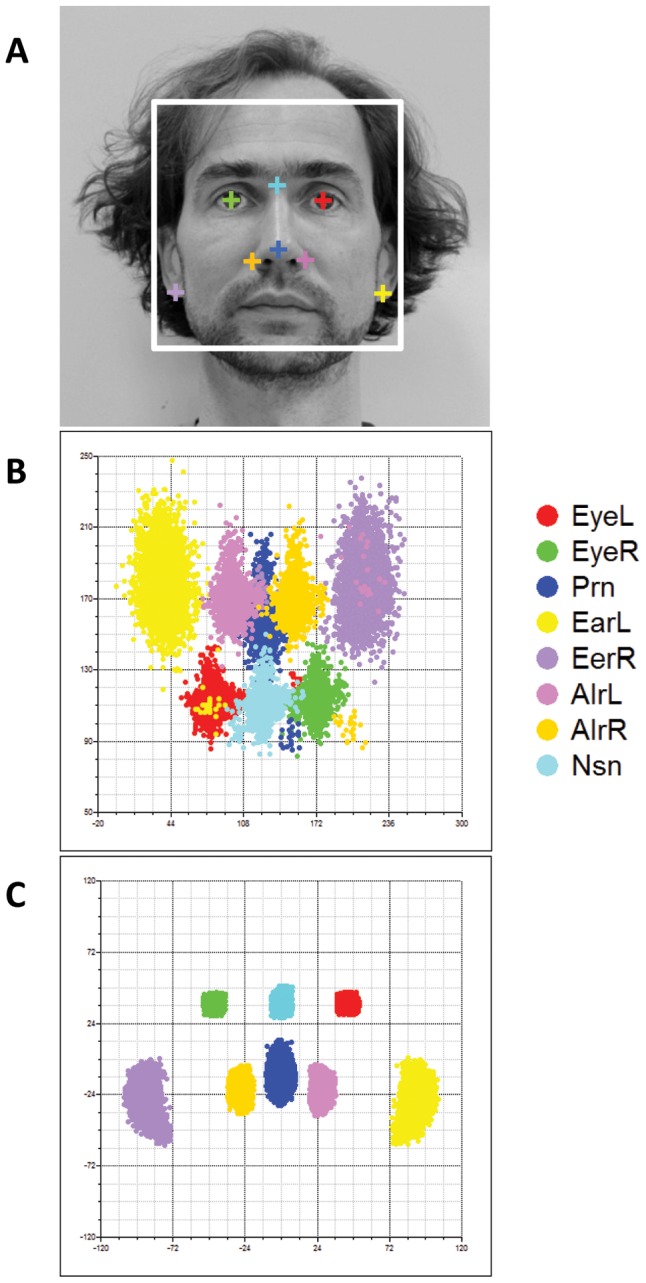
Facial landmarks from 2D portrait photos. Eight facial landmarks extracted from a 2D portrait photo of one of the authors (MK) to illustrate facial shape phenotyping in the 2D portrait photos (A). Landmark configurations in 2D photos from 3,503 individuals from the replication cohorts BLTS and TwinsUK before (B), and after (C) full PS. Note that raw landmarks in B appeared to be upset-down of a face, which were rotated by 180° in C.

In addition to the direct replication of MRI-based phenotypes in the SYS cohort, we sought further supporting evidence of association in a combined data set of two additional samples from the UK (TwinsUK, N = 1,366) and Australia (BLTS, N = 2,137) where we localized eight facial landmarks on 2D portrait photos (illustrated in Figure 5A). Raw landmark coordinates showed significant differences not only in position and orientation but also in size (Figure 5B); we thus used full PS including rescaling to remove these differences (Figure 5C). Note that the inter-landmark distances from 2D photos, with or without rescaling, do not represent the absolute distance in terms of millimeters, as those from 3D MRIs do. Furthermore, the fact that 2D data in general miss a complete dimension may potentially lead to false negative replications. Note also that the twin correlations for the inter-landmark distances, estimated based on 2D photos, were in general much lower than those from 3D MRIs (*r* = 0.42 in 218 MZ pairs, *r* = 0.16 in 533 DZ pairs; TwinsUK and BLTS combined sample), indicating that these phenotypes were more noisy than those derived from the 3D images. In spite of these limitations, we observed nominally significant associations for approximately the same phenotypes for 2 of the 5 loci identified from our GWAS, *TP63* and *C5orf50* (P<0.05; Table 2). The associations at these 2 loci were also observed for multiple phenotypes, i.e. P<0.05 for 17.9–21.4% of all 28 inter-landmark distances (Table 2). Thus, except for *PRDM16* and *PAX3*, all loci identified with genome-wide significance in the discovery cohorts were replicated in 3D MRI (SYS) or 2D photo (TwinsUK, BLTS) analyses. For *PAX3* there was a significant association between rs974448 and the distance between the eyeballs in the 2D data (beta = 0.30, se = 0.15, P = 0.045, data not shown, note in Table 2 the results for *PAX3* are shown for Eye-Nsn phenotypes). In our discovery phase GWAS a total of 102 SNPs at 29 distinct loci showed significant or suggestive evidence of association (P<5×10^−7^) with various facial phenotypes (Table S5). We provide raw genotype and respective phenotype data for all SNPs that revealed genome-wide significant and suggestive evidence (Table S6) to make our most important findings publically available to other researchers who may wish to explore them further.

Finally, we re-investigated the potential association between SNPs previously involved in NSCL/P and normal facial shape variation in our discovery cohorts (Table 3). For this purpose, we tested associations between facial phenotypes and 11 SNPs ascertained in our previous candidate gene study [20], originally discovered in previous GWAS on NSCL/P [35], [36], [37], [38]. Five SNPs at 4 candidate NSCL/P loci were significantly associated with normal facial phenotypes even after a strict Bonferroni correction of multiple testing of all 48 correlated phenotypes (Table 3). These included rs7590268 at 2p21, rs16903544 and rs987525 at 8q24, rs9574565 at 13q31, and rs227731 at 17q22. All these SNPs were also associated with over 10% of 48 facial phenotypes at P<0.05, where rs987525 at 8q24 was associated with over half of the studied phenotypes (52.08%, Table 3). In addition, the SNP rs642961 at chromosome 1q32 was associated with 27.1% of the studied phenotypes at P<0.05, although the minimal P value was not significant after the over-conservative Bonferroni correction. The SNP rs1258763 at 15q13 was nominally significantly associated with nose-width (P = 0.03 for AlrR-AlrL, Table 3), although not significant after the Bonferroni correction. These findings strongly suggest that genetic variants involved in NSCL/P also influence normal facial shape variation.

**Table 3:** NSCL/P cleft-associated SNPs in association with normal facial shape variation (N = 5,388).

SNP	Chr	Position	Eff	Alt	FreqEff	CallRate	Sign%	Trait	Beta	Se	minP	Bonferroni	AlrR-AlrL	ZygR-ZygL
rs560426	1p21	94326026	C	T	0.47	0.99	0.0	ZygL-EyeL	−0.09	0.06	9.88E-02	1.000	0.532	0.869
rs642961	1q32	208055893	A	G	0.21	1.00	27.1	EyeR-Prn	−0.27	0.09	4.80E-03	0.231	0.121	0.144
rs7590268	2p21	43393629	G	T	0.22	0.99	10.4	PC11	−0.12	0.03	7.19E-05	**0.003**	0.734	0.082
rs16903544	8q24	129714416	C	T	0.10	0.98	35.4	ZygR-AlrL	0.42	0.12	4.48E-04	**0.022**	0.193	**0.019**
rs987525	8q24	130015336	A	C	0.23	1.00	52.1	ZygL-EyeR	−0.32	0.09	5.89E-04	**0.028**	0.132	**0.003**
rs7078160	10q25	118817550	T	C	0.16	0.15	–	–	–	–	–	–	–	–
rs9574565	13q31	79566875	T	C	0.24	0.84	16.7	EyeR-Prn	0.34	0.10	8.74E-04	**0.042**	0.628	0.823
rs1258763	15q13	30837715	C	T	0.32	0.99	2.1	AlrR-AlrL	−0.12	0.06	3.27E-02	1.000	**0.033**	0.849
rs17760296	17q22	51970616	G	T	0.16	0.99	4.2	PC4	−0.19	0.07	5.70E-03	0.274	0.789	**0.032**
rs227731	17q22	52128237	G	T	0.44	0.98	12.5	PC6	0.15	0.04	7.96E-05	**0.004**	**0.045**	0.638
rs13041247	20q12	38702488	C	T	0.39	1.00	2.1	AlrR-Sbn	−0.08	0.03	1.93E-02	0.929	0.069	0.143

Sign%, the percentage of P values<0.05 in 48 phenotypes.

Trait, the phenotype which showed the minimal P value.

Bonferroni, corrected by 48 multiple testing.

AlrR-AlrL, P value for nose-width.

ZygR-ZygL, P value for bizygomatic distance.

## Discussion

We identified five independent loci at 1p36.23-p33, 2q35, 3q28, 5q35.1, and 10q24.3 consisting of common DNA variants associated with normal facial shape phenotypes in individuals of European ancestry. Candidate genes at these loci include *PRDM16* (PR domain containing 16), *PAX3* (paired box 3), *TP63* (tumor protein p63), *C5orf50* (chromosome 5 open reading frame 50), and *COL17A1* (collagen, type XVII, alpha 1). In addition to our GWA findings, we confirmed links between NSCL/P cleft associated SNPs at 2p21, 8q24, 13q31, and 17q22 and normal human facial shape variation based on a candidate gene approach.

From a statistical perspective, the most robust result was the one at the *PAX3* locus. We identified this gene in our discovery GWAS, a finding independent of, but consistent with, a recent GWAS [34]. Although the association with the same set of phenotypes failed replication in our replication cohorts, the identification of the same locus at the genome-wide significant level from completely independent GWASs cannot be coincidence. This provides strong statistical evidence that the *PAX3* gene is indeed involved in facial morphology. The signal observed at *C5orf50* at 5q35.1 in association with the nasion position was successfully replicated in both SYS using 3D MRI derived phenotypes under similar (buit not identical) methodology as well as in a combined sample of TwinsUK and BLTS using 2D photograph derived phenotypes. The association signal observed at *COL17A1* was replicated only in SYS (3D MRI) and that at *TP63* was replicated only in TwinsUK and BLTS (2D photos). The signal observed at *PRDM16* was not replicated in our replication cohorts. The failure in replication for some of our GWAS findings may be explained by physical limitations in our replication cohorts as discussed in detail below.

Three of the five loci identified in this study have previously been shown to play an essential role in craniofacial development; in particular, they have been implicated in orofacial clefting phenotypes in mice or humans. *PAX3* encodes a developmentally important transcription factor expressed in neural crest cells, a multipotent cell population contributing to most differentiated cell types in the vertebrate face. In humans, *PAX3* is one of six genes mutated in Waardenburg syndrome, which is characterized by a range of neural crest related phenotypes including minor facial dysmorphism manifest as a broad nasal root and an increased distance between the medial canthi or corners of the eye (telecanthus) [39]. Studies in mice have demonstrated that failure to down regulate *PAX3* during neural crest differentiation leads to cleft palate, due to inhibitory effects on osteogenesis [40]. Of particular relevance to this study, a recent GWAS detected an association between *PAX3* and position of the nasion [34].

*PRDM16* was previously identified as a SMAD binding partner; it is thought to act in downstream mediation of TGFβ signaling in developing orofacial tissues [41]. Consistent with this role, *PRDM16* is expressed in both the primary and secondary palate and the nasal septum in mouse embryos [41]. Functional studies in mice confirm a role in craniofacial development, with an *N*-ethyl-*N*-nitrosourea-induced mutation in *PRDM16* resulting in cleft palate and other craniofacial defects including mandibular hypoplasia [42]. Moreover, variants at the human *PRDM16* locus have been implicated in NSCL/P [42].

*TP63* encodes a transcription factor belonging to the p53 family, and plays important roles in orchestrating developmental signaling and epithelial morphogenesis. Heterozygous mutations in human *TP63* are associated with a number of allelic syndromes characterized by orofacial defects, including Ectrodactyly-Ectodermal dysplasia-Cleft lip/palate and Ankyloblepharon-Ectodermal dysplasia-Clefting [43]. Furthermore, *TP63* has been implicated in human NSCL/P [44], and null mice recapitulate the human orofacial clefting phenotypes [45].

The remaining two loci identified in the discovery sample and replicated in both the SYS and TwinsUK & BLTS samples have no previously known direct involvement in craniofacial development to date. *C5orf50* at 5q35.1 is predicted to encode an uncharacterized transmembrane protein, which lies within a 1.24 Mb duplicated region in a patient with preaxial polydactyly and holoprosencephaly (HPE), a defect in development of the forebrain and midface [46]. The most likely candidate in the duplicated region is *FBXW11*, a gene with links to sonic hedgehog signaling, the main pathway affected in HPE [47]. It is therefore possible that variants at the *C5orf50* locus influences craniofacial patterning through effects on *FBXW11* expression, although it is also feasible that the protein encoded by *C5orf50* has a novel, and more direct, effect on the face. Mutations in *COL17A1* cause Junctional epidermolysis bullosa (JEB), a genetic blistering condition [48], however, there is no evidence to date for a direct involvement of this gene in craniofacial morphogenesis. Our data suggest that *COL17A1* may play an as yet undefined role in patterning facial tissues. However, the association signal observed for *COL17A1* at 10q24.3 spans a 300 kb region (105.7 Mb–106 Mb) and also harbors other genes including *SLK*, *C10orf78* and *C10orf79*. Thus, we cannot exclude the possibility that these genes are responsible for the observed association.

Many medical-genetic syndromes show a clear connection between genetic alteration and typical facial gestalt [49], hence genes involved in affected individuals may also contribute to normal variations in facial shape. Our previous study of 11 NSCL/P associated SNPs [20] showed some borderline significance for association with nose-width and bizygomatic distance but inconsistent effect was observed in two populations studied (Rotterdam and Essen). This discrepancy may be partially explained by sample size and different sources of facial phenotype, namely 3D MRI in the Rotterdam Study and 2D portrait photos in Essen. In the 2D facial pictures in the Essen sample, for instance, the bizygomatic distance was defined indirectly by neighboring landmarks of the face [50]. In the current study using 3D MRI data in a larger sample (N = 5,388), multiple SNPs showed significant association with multiple facial phenotypes, even after an over-conservative Bonferroni correction. Thus, in the present study we established clear links between NSCL/P associated SNPs at 2p21, 8q24, 13q31, and 17q22 and normal facial shape phenotypes, including nose width and facial width, in general populations of European descent. This is in line with previous evidence showing that unaffected relatives of NSCL/P cleft patients have wider upper faces and noses than unrelated individuals [51], [52]. Together with our GWAS findings at three loci previously known to play a role in orofacial clefting, our data strongly suggest that genetic variants associated with NSCL/P also influence normal facial shape variation.

This study is not without limitations. The limited number of landmarks used in this study cannot capture the full range of the complex 3D shape variation in the face. This is partly due to the physical limitation of our MRI data that miss the lower part of the face and partly due to other factors such as partial incompatibility of 3D and 2D image sources for phenotype extraction. Consequently, some of the significant associations based on 3D distances could not be tested in the 2D photo analysis. For instance, the zygion landmarks available in 3D MRI could not be successfully derived in 2D photos. Further, the precision of phenotypes derived from 2D photos is expected to be lower than that from 3D MRIs. This is indicated by the lower twin correlations in 2D photos than in 3D MRI. Phenotypic noise in 2D photos may arise from slight differences in face orientation, an effect that cannot be removed by PS without measuring the 3rd coordinate. Furthermore, different image sizes and pixel resolutions in 2D photos may also influence the phenotyping results. Thus, we used the 2D photo analysis to provide supporting information but cannot be considered as an exact replication. Another concern is that the facial landmarks from the SYS cohort were derived in a previous study [22] based on slightly different definitions compared with the ones from the five discovery cohorts. This may potentially lead to some heterogeneity in replication results. Furthermore, the SYS cohort consists of adolescents. Some of us have shown previously that several facial features continue to change during the male (but not female) adolescents [23]. This study included only samples of European ancestry, which reduces the potential risk of false positive findings due to systematic genetic differences between different populations. On the other hand, further investigation in world-wide samples is required to generalize our findings to populations other than Europeans, and to investigate the genetic basis of particular facial phenotypes that are absent from Europeans. In addition, we have only focused on common variants (MAF>3%); further investigations of less common and/or quite rare variants as for instance available from next generation sequencing data may provide a more complete figure on the genetic basis of facial morphology.

In spite of these limitations we have been able to demonstrate that a phenotype as complex as human facial morphology can be successfully investigated via the GWAS approach with a moderately large sample size. Three of the five loci highlighted here map to known developmental genes with a previously demonstrated role in craniofacial patterning, one of which has been unequivocally associated with nasion position in a recent independent GWAS [34]. The remaining two loci map to or close to *C5orf50* and *COL17A1*, neither of which have previously been implicated in facial development. The associated DNA variants may either affect neighboring genes, or alternatively identify *C5orf50*, and *COL17A1* as potential new players in the molecular regulation of facial patterning. Overall, we have uncovered five genetic loci that contribute to normal differences in facial shape, representing a significant advance in our knowledge of the genetic determination of facial morphology. Our findings may serve as a starting point for future studies, which may test for allele specific expression of these candidate genes and re-sequence their coding regions to identify possible functional variants. Moreover, our data also highlight that the high heritability of facial shape phenotypes (as estimated here and elsewhere), similar to that of adult body height [53], involves many common DNA variants with relatively small phenotypic effects. Future GWAS on the facial phenotype should therefore employ increased sample sizes as this has helped to identify more genes for many other complex human phenotypes such as height [53] and various human diseases. Combined with the emerging advances in 3D imaging techniques, this offers the poteintial to advance our understanding of the complex molecular interactions governing both normal and pathological variations in facial shape.

## Materials and Methods

### Rotterdam Study (RS), The Netherlands

The RS is an ongoing population-based prospective study including a main cohort RS-I [54] and two extensions RS-II and RS-III [55], [56], including 15,000 participants altogether, of whom 12,000 have GWAS data. Collection and purification of DNA have been described in detail previously [57]. A subset of participants were scanned on a 1.5 T General Electric MRI unit (GE Healthcare, Milwaukee, WI, USA), using an imaging protocol including a 3D T1-weighted fast RF gradient recalled acquisition in steady state with an inversion recovery prepulse. The following parameters were used: 192 slices, a resolution of 0.49×0.49×0.8 mm^3^ (up sampled from 0.6×0.7×0.8 mm^3^ using zero padding in the frequency domain), a repetition time (TR) of 13.8 ms, an echo time (TE) of 2.8 ms, an inversion time (TI) of 400 ms, and a flip angle of 20°. More details on image acquisition can be found elsewhere [58]. The Medical Ethics Committee of the Erasmus MC, University Medical Center Rotterdam, approved the study protocol, and all participants provided written informed consent. Principal components analysis of SNP microarray data was used to identify ancestry outliers. These were removed before further analyses, and the present sample is of exclusively northern/western European origin. The current study included 3,215 RS participants who had both SNP microarray data and 3D MRI. These participants were considered here as two cohorts (RS1 N = 2,470 and RS2 N = 745) as they were scanned and genotyped at different times.

### Brisbane Longitudinal Twin Study (BLTS) and Queensland Twin Imaging Study (QTIMS), Australia

Adolescent twins and their siblings were recruited over a period of sixteen years into the BLTS at 12, 14 and 16 years, as detailed elsewhere [59] and as young adults into the QTIMS [60], [61]. The present study includes a sub-sample of 545 young adults (aged 20–30 years, M = 23.7±2.3 years; 79 MZ and 90 DZ pairs, 110 unpaired twins, and 97 singletons, from a total of 332 families) from QTIMS with 3D MRIs, and 2,137 adolescents (aged 10–22 years, M = 15.6±1.5 years; 311 MZ and 530 DZ pairs, 44 unpaired twins and 411 singletons, from a total of 1,038 families) from BLTS with 2D portrait photos. 3D T1-weighted MR images were collected at the Centre for Advanced Imaging, University of Queensland, using a 4T Bruker Medspec whole body scanner (Bruker Medical, Ettingen, Germany) [61]. 2D portrait photos were taken from a distance of 1–2 meters for identification, with no specific instruction given to smile. Those who had both 3D MRI scans and 2D photos were included in discovery GWAS and excluded from the replication analysis in 2D photos. Over 70% were digital with the remainder being scanned from high quality film. The study was approved by the Human Research Ethics Committee, Queensland Institute of Medical Research. Informed consent was obtained from all participants (or parent/guardian for those aged less than 18 years).

### Study of Health In Pomerania (SHIP), Germany

The SHIP is a longitudinal population based cohort study assessing the prevalence and incidence of common, population relevant diseases and their risk factors with examinations at baseline (SHIP-0, 1997–2001), 5-year-follow-Up (SHIP-1, 2002–2006) and an ongoing 10-year-follow-Up (SHIP-2, 2008–2012) [62], [63]. Data collection from the baseline sample included 4,308 subjects. A new cohort targeted 5,000 participants (SHIP-TREND) has been started parallel to the SHIP-2-Follow-Up. In addition to the baseline examinations, participants of SHIP-2 and SHIP-TREND also had a whole-body MRI scan [64]. MRI examinations were performed on a 1.5T MR imager (Magnetom Avanto; Siemens Medical Systems, Erlangen, Germany). Head scans were taken with an axial ultra-fast gradient echo sequence (T1 MPRage, TE 1900.0, TR 3.4, Flip angle 15°, 1.0×1.0×1.0 mm voxel size). The present study includes 797 SHIP as well as 831 SHIP-TREND participants which had both SNP genotyping data and 3D MRI. The medical ethics committee of University of Greifswald approved the study protocol, and oral and written informed consents were obtained from each of the study participants.

### Saguenay Youth Study (SYS), Canada

Adolescent sibpairs (12 to 18 years of age) were recruited from a French-Canadian population with a known founder effect living in the Saguenay-Lac-Saint-Jean region of Quebec, Canada in the context of the ongoing Saguenay Youth Study [65]. Local ethics committee approved the study; the parents and adolescent participants gave informed consent and assent, respectively. MRI was acquired on a Phillips 1.0-T magnet using the following parameters: 3D radio frequency spoiled gradient-echo scan with 140–160 slices, an isotropic resolution of 1 mm, TR 25 ms, TE 5 ms, and flip angle of 30°. Outlying individuals including those with putative Indigenous American admixture were excluded based on genetic outlier analysis [66]. The current study contains 568 adolescents with MRI and SNP data.

### St. Thomas' UK Adult Twin Registry (TwinsUK), United Kingdom

The TwinsUK cohort is unselected for any disease and is representative of the general UK population [67]. All were volunteers, recruited through national media campaigns. Written informed consent was obtained from every participant. Population substructure and admixture was excluded using eigenvector analysis on SNP microarray data. The current study included 1,366 individuals with 2D portrait photos and SNP microarray data.

### Facial landmarks from 3D head MRI

In our discovery cohorts, since the lower part of the face was not available from the MRIs, we focused on nine landmarks of the upper face (Figure 1). These included Right (ZygR) and left (ZygL) zygion: the most lateral point located on the cortex of the zygomatic arches; right (EyeR) and left (EyeL) eyeball: the middle point of the eyeball; right (AlrR) and left (AlrL) alare: the most lateral point on the surface of the ala nasi; nasion (Nsn): the skin point where the bridge of the nose meets the forehead; pronasale (Prn): the most anterior tip of the nose; subnasale (Sbn): the point where the base of the nasal septum meets the philtrum. Although these landmarks provide only a very sparse representation of the facial shape, they cover most prominent facial features and are easy to interpret and compare to other studies [22], [34], [51]. Furthermore, these landmarks could be measured with higher accuracy from images than most semilandmarks [22].

The coordinates of these nine landmarks were derived with an automated technique as described previously [20], which uses image registration to transfer predefined landmarks from a limited set of annotated images to an unmarked image. The manual annotation was based on landmark definitions from the anthropology literature [68], which were adapted for application to T1-weighted MR images of the head. None of the MRI showed distortions in a visual inspection. Furthermore, the automatically localized landmark positions were robust against the number of samples included. The test-retest correlations based on a subset of 40 subjects from QTIMS who were scanned twice were high (r>0.99).

In the SYS cohort, in total 56 facial landmarks were available from a previous quantitative analysis of craniofacial morphology using 3D MRI [22]. In brief, an average MRI was constructed using non-rigid image registration. The surface of this average image represents the mean facial features and was then annotated with 56 landmarks and semi-landmarks. These landmarks were then warped using the nonlinear transformation that maps each subject to the average. This allows for automatic identification of the different craniofacial landmarks.

### Facial landmarks from 2D portrait photos

We defined eight landmarks in 2D portrait photos that approximately correspond to the respective landmarks ascertained from our 3D MRIs. These include EyeL, EyeR, Prn, AlrL, AlrR, Nsn, earlobe left (EarL), and earlobe right (EarR) (Figure 5A). Note the Sbn, ZygL and ZygR landmarks available in 3D MRIs could not derived in 2D photos. We developed an algorithm to locate these landmarks in 2D portrait images and implemented it in an in-house C++ program. Briefly, the algorithm first recognizes the face layout within an image by matching a face template. It then recognizes eyes, nose, and ears by matching corresponding templates. The template matching routines were based on external open source computer vision library, OpenCV 2.3.1 (http://sourceforge.net/projects/opencvlibrary/). The automatically identified landmarks were then manually adjusted by 5 research assistants on a standard computer screen.

### Facial shape phenotypes

We used un-scaled PS, or partial PS [24], [69], to superimpose the landmarks from the 3D MRIs in the discovery cohorts onto a consensus 3D Euclidean space. Unlike full PS, partial PS only re-positions and re-orientates but does not rescale the landmark configurations; thus, it has no effect on the Euclidean distances between landmarks as measured in terms of millimeters from MRIs. Keeping the absolute inter-landmark distances allows us to interpret the association results more directly. Furthermore, the full PS has been criticized for introducing artificial correlations between landmarks [70]. We considered the centroid size as a measurement of face size, and it was significantly correlated with absolute head volume (*r* = 0.95). We derived 11 principal components (PCs) from the superimposed landmarks, each explaining at least 1% of the total phenotypic variance. We also derived 36 Euclidean distances between all pairs of landmarks. Thus 48 phenotypes were included in our GWAS, including centroid size, 11 PCs, and 36 inter-landmark distances. All phenotypes were approximately normally distributed and outliers (>3sd) were removed. Deformation approaches including the use of transformational grids provide an alternate way to study shape difference. Thin plate splines (TPS) [27] depicts the deformation geometrically, where the total deformation is decomposed into several orthogonal components to localize and illustrate the shape differences. We used TPS to illustrate the facial shape differences between males and females using the tpsgrid function in R library shapes.

For 3D MRI data in SYS, we used the 56 landmarks derived in a previous study and calculated 1,540 Euclidean distances between all pairs of landmarks. These distances were considered as phenotypes in our replication analysis of GWAS findings. We also chose a subset of nine landmarks most closely resembled those used for the current study for exact replication.

Since the size of the face vary substantially between 2D portrait images, we used the full PS [24] to also remove the scaling differences between landmark configurations. Note that the inter-landmark distances from 2D photos do not represent the absolute distances in terms of millimeters regardless of whether full or partial PS was used. After superimposition, we calculated 28 Euclidean distances between all pairs of the 8 landmarks, which were considered as phenotypes in the replication analysis. The PS analyses were performed with CRAN package shapes developed by Ian Dryden [30].

### Heritability analysis

By clarifying which facial features are under strong genetic control, we should be better able to identify specific genes that influence facial variation. Heritability estimates are also important indicators of the phenotype quality. Using QTIMS (79 MZ pairs, 90 DZ pairs) heritability analysis was carried out in Mx [71] using full information maximum likelihood estimation of additive genetic variance (i.e. heritability), common environmental variance, and unique environmental variance. Sex and age were included as covariates. Phenotypic correlations were estimated in BLTS (311 MZ pairs, 90 DZ pairs) and in TwinsUK (93 MZ pairs, 352 DZ pairs) where the facial shape phenotypes were derived from 2D photos.

### Genotyping, imputation, quality control

Details of SNP microarray genotyping, quality control and genotype imputation are described in prior GWAS conducted in RS [16], QTIMS and BLTS [72], SHIP [62], SYS [73], and TwinsUK [74]. In brief, DNA samples from the RS, BTNS, SYS and TwinsUK cohorts were genotyped using the Human 500–610 Quad Arrays of Illumina and samples from SHIP were genotyped using the Genome-Wide Human SNP Array 6.0 of Affymetrix and HumanOmni2.5 of Illumina, respectively. Genotyping of the SHIP-TREND probands (n = 986) was performed using the Illumina HumanOmni2.5-Quad, which has not been reported previously and described here as follows. DNA from whole blood was prepared using the Gentra Puregene Blood Kit (Qiagen, Hilden, Germany) according to the manufacturer's protocol. Purity and concentration of DNA was determined using a NanoDrop ND-1000 UV-Vis Spectrophotometer (Thermo Scientific). The integrity of all DNA preparations was validated by electrophoresis using 0.8% agarose-1x TBE gels stained with ethidium bromide. Subsequent sample processing and array hybridization was performed as described by the manufacturer (Illumina) at the Helmholtz Zentrum München. Genotypes were called with the GenCall algorithm of GenomeStudio Genotyping Module v1.0. Arrays with a call rate below 94%, duplicate samples as identified by estimated IBD as well as individuals with reported and genotyped gender mismatch were excluded. The final sample call rate was 99.51%. Imputation of genotypes in the SHIP-TREND cohort was performed with the software IMPUTE v2.1.2.3 against the HapMap II (CEU v22, Build 36) reference panel. 667,024 SNPs were excluded before imputation (HWE p-value≤0.0001, call rate ≤0.95, monomorphic SNPs) and 366 SNPs were removed after imputation due to duplicate RSID but different positions. The total number of SNPs after imputation and quality control was 3,437,411. The genetic data analysis workflow was created using the Software InforSense. Genetic data were stored using the database Caché (InterSystems). After SNP imputation to the HapMap Phase II CEU reference panel (Build 36) and quality control, 2,558,979 autosomal SNPs were common in all discovery cohorts and used for analyses.

### GWAS and replication

We conducted discovery phase GWAS in a combined set of all discovery cohorts (RS1, RS2, QTIM, SHIP, SHIP-Trend) for 48 facial shape phenotypes. Imputed GWAS data in all discovery cohorts were merged according to the positive strand. We tested 2,558,979 autosomal SNPs with linear regression (adjusted for sex, age, EIGENSTRAT-derived ancestry informative covariates [75], plus any additional ancestry informative covariates as appropriate) in GenABEL [76]. The centroid size was adjusted in the analysis of inter-landmark distances. SNPs with MAF<3%, overall call rate <95%, and HWE P<1×10^−3^ were not considered for report. Genomic inflation factors were estimated in range 1.0–1.03 for all studied phenotypes. The observed P-values were Q-Q plotted against the expected P-values at −log10 scale. We considered the traditional threshold of 5×10^−8^ as being genome-wide significant since many phenotypes were highly correlated. All SNPs in this study were annotated based on NCBI build 36.3.

The linear modeling used here separately analyzes each facial phenotype. It is also possible to derive a global P-value for testing the shape difference between different genotype groups using other approaches, such as the Euclidean Distance Matrix Analysis (EDMA) [28], [29] and the multivariate analysis of variance (MANOVA) [77]. The EDMA computes a score of the maximal ratio [28] or difference [29] between the mean shapes estimated in two groups. Since this score does not follow a known distribution, the statistical significance is derived by bootstrapping all landmark configurations. In the context of GWAS, this bootstrap procedure should be conducted for every SNP, which turned out to be computationally heavy when we attempted to implement it at the genome-wide scale. In addition, EDMA is less flexible than linear modeling when the effects of covariates are to be adjusted and when more than two genotype groups are to be compared. The MANOVA is a classic statistical method for analysis of multiple correlated response variables, which has been shown to be useful in GWAS [78]. We implemented MANOVA for GWAS in R and conducted a GWAS for the residuals of the 11 facial shape PCs after regressing out the effect of sex, age, and population stratification. However, no significant signal (P<5×10^−8^) was observed for SNPs with MAF>3% (results not shown).

All SNPs with P values<5×10^−8^ in our discovery phase GWAS were sought for replication in SYS, TwinsUK, and BLTS. Promising SNPs were tested for association with 1,054 inter-landmark distances in SYS and 28 inter-landmark distances in a combined sample of TwinsUK and BLTS assuming additive allelic effect adjusted for sex and age using MERLIN [79], which also takes into account family relationships. We report the association results for the same phenotypes as discovered in GWAS as exact replication. In addition, for each SNP we report the percentage of significantly (P<0.05) associated phenotypes, which is expected to be lower than 5% under the null hypothesis of no association. For the analysis of 11 NSCL/P associated SNPs in our discovery cohorts, we additionally Bonferroni corrected the P values for 48 correlated phenotypes since no specific facial phenotypes were selected for replication.

## Supporting Information

Figure S1 Thin plate spline deformation illustrating facial shape differences in males compared to females in discovery cohorts (N = 5388). The pixel information obtained from the mean shape of males was mapped to that of females. The deformed images illustrate the difference between the mean shpae of males (the curved plates) compared to that of females (imaginary flat plates). A. a 3D view of the mean facial shape of all individuals in the discovery cohorts before deformation; B. side projection of the deformed grid; C. front projection of the deformed grid; D. up-down projection of the deformed grid.
(TIF)

Table S1 Correlation between PC and Euclidean distances in discovery cohorts. The color shade is independent between columns.
(XLSX)

Table S2 Correlation matrix between pair-wise Euclidean distances and size in discovery cohorts.
(XLSX)

Table S3 Effect of sex and age on facial shape in discovery cohorts. Distances are presented in millimeters. P values were adjusted for centroid size.
(XLSX)

Table S4 Heritability of facial shape phenotypes derived from 3D MRI in QTIMS. Twin correlations and proportions of variance due to additive genetic (A), common environmental (C), and unique environmental (E) influences, shown with 95% confidence intervals (age and sex adjusted).
(XLSX)

Table S5 SNPs (n = 102) associated (P<5e-7) with facial shape phenotypes in discovery phase GWAS. Trait, the phenotype for which the minimal P value was obtained. MinP, the minimal P value.
(XLSX)

Table S6 Raw genotype and respective phenotype data for all SNPs that revealed genome-wide significant and suggestive evidence.
(XLSX)
