# Supplementary figures and images for: A Genome-Wide Association Study Identifies Five Loci Influencing Facial Morphology in Europeans
Source: PLoS Genet. 2012 Sep 13;8(9):e1002932. doi: 10.1371/journal.pgen.1002932 (PMC3441666; doi:10.1371/journal.pgen.1002932)

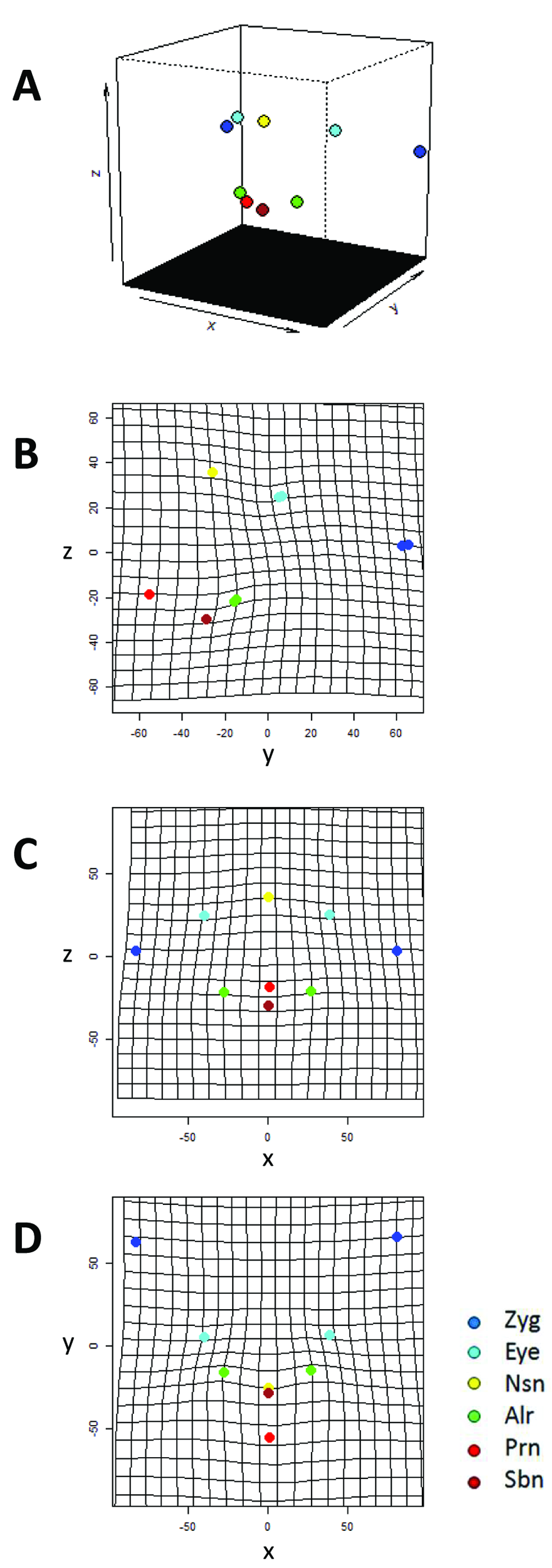

Supplement: Figure S1 — Thin plate spline deformation illustrating facial shape differences in males compared to females in discovery cohorts (N = 5388). The pixel information obtained from the mean shape of males was mapped to that of females. The deformed images illustrate the difference between the mean shpae of males (the curved plates) compared to that of females (imaginary flat plates). A. a 3D view of the mean facial shape of all individuals in the discovery cohorts before deformation; B. side projection of the deformed grid; C. front projection of the deformed grid; D. up-down projection of the deformed grid. (TIF) [file pgen.1002932.s001.tif]
